# Stress-induced inhibition of mRNA export triggers RNase III-mediated decay of the *BDF2* mRNA

**DOI:** 10.1261/rna.078880.121

**Published:** 2021-12

**Authors:** Charles Wang, Keaton Barr, Dean Neutel, Kevin Roy, Yanru Liu, Guillaume F. Chanfreau

**Affiliations:** 1 Department of Chemistry and Biochemistry, UCLA, Los Angeles, California 90095, USA; 2 Molecular Biology Institute, UCLA, Los Angeles, California 90095, USA

**Keywords:** Rnt1p, RNase III, Bromodomain, mRNA export, stress

## Abstract

The expression of bromodomain-containing proteins that regulate chromatin structure and accessibility must be tightly controlled to ensure the appropriate regulation of gene expression. In the yeast *S. cerevisiae*, Bromodomain Factor 2 (*BDF2)* expression is extensively regulated post-transcriptionally during stress by RNase III-mediated decay (RMD), which is triggered by cleavage of the *BDF2* mRNA in the nucleus by the RNase III homolog Rnt1p. Previous studies have shown that RMD-mediated down-regulation of *BDF2* is hyperactivated in osmotic stress conditions, yet the mechanisms driving the enhanced nuclear cleavage of *BDF2* RNA under these conditions remain unknown. Here, we show that RMD hyperactivation can be detected in multiple stress conditions that inhibit mRNA export, and that Rnt1p remains primarily localized in the nucleus during salt stress. We show that globally inhibiting mRNA nuclear export by anchoring away mRNA biogenesis or export factors out of the nucleus can recapitulate RMD hyperactivation in the absence of stress. RMD hyperactivation requires Rnt1p nuclear localization but does not depend on the *BDF2* gene endogenous promoter, and its efficiency is affected by the structure of the stem–loop cleaved by Rnt1p. Because multiple stress conditions have been shown to mediate global inhibition of mRNA export, our results suggest that the hyperactivation of RMD is primarily the result of the increased nuclear retention of the *BDF2* mRNA during stress.

## INTRODUCTION

The covalent modification of histones is a major epigenetic mechanism that regulates the accessibility of DNA for damage repair, transcriptional activation or repression and heterochromatin formation (Lawrence et al. 2016). Histones acetylation is a well-studied covalent modification that is generally associated with transcriptional activation (Kurdistani and Grunstein 2003). Bromodomain-containing proteins bind to acetylated histones and recruit various proteins to alter gene expression (Josling et al. 2012). As such, they are key players in transcriptional regulation, and bromodomains have recently emerged as therapeutic targets in oncogenesis and various pathological conditions (Morgado-Pascual et al. 2019; Gilan et al. 2020). Bromodomain factor 2 (Bdf2p) is one of the two bromodomain-containing proteins found in the yeast *S. cerevisiae* that recognizes acetylated lysines on histones. Bdf2p was found to establish heterochromatin boundaries and regulates the yeast salt stress response, although the specific mechanisms that govern these processes remain elusive (Fu et al. 2013). Bdf2p is not essential for growth in *S. cerevisiae*. However, strains lacking both Bdf2p and Bdf1p are inviable (Matangkasombut et al. 2000), suggesting a partial redundancy between the functions of the two proteins. Indeed, *BDF2* overexpression can rescue the salt sensitivity and mitochondrial dysfunction of *bdf1*Δ mutants (Fu et al. 2013). This functional redundancy is further confirmed by the observation that Bdf2p can occupy sites normally bound by Bdf1p in mutants lacking Bdf1p (Durant and Pugh 2007). The absence of Bdf1p also increases the basal expression of *BDF2* (Fu et al. 2013; Volanakis et al. 2013). Interestingly, Bdf2p has been found to interact with the general Pol.II transcription factor TFIID, implicating a possible broader role in regulating transcription (Matangkasombut et al. 2000; Fu et al. 2013). These observations show that a precise regulation of *BDF2* is necessary to balance its expression relative to that of *BDF1* and during stress.

**FIGURE 1.**
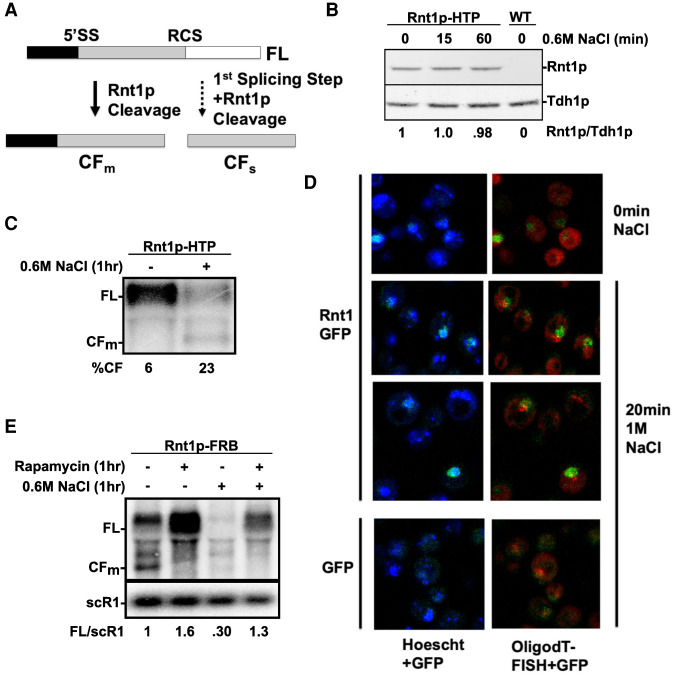
Rnt1p protein levels and localization remain unchanged during after salt stress. (*A*) Schematic representation of the cleavage of the *BDF2* mRNA by Rnt1p and by the spliceosome. Shown are the two 5′-cleavage products generated by Rnt1p-mediated decay (RMD; CFm) or by spliceosome mediated decay and RMD (CFs). These cleavage products are typically degraded by nuclear exonucleases but can be detected by inactivation of Rrp6p. The 3′ fragments are not represented. (*B*) Western blot analysis of Rnt1p harboring an HTP carboxy-terminal tag (Granneman et al. 2009). Cells were treated with or without 0.6 M NaCl for the indicated times. Tdh1p was used as a loading control. An untagged wild-type strain was used as a negative control. Numbers *below* the blot show the average of three independent biological replicates. (*C*) Northern blot analysis of *BDF2* in the Rnt1-HTP tagged strain. Shown is a northern blot of *BDF2* using a probe spanning the *BDF2* initiation codon until the Rnt1p cleavage site (RCS; Fig. 1A) of RNAs extracted from a Rnt1-HTP tagged strain grown in normal medium or after a shift to NaCl (0.6 M, 1 h). The ncRNA scR1 was used as loading control. Numbers indicate the % of the *BDF2* cleavage fragment CFm relative to all *BDF2* transcripts. Numbers are the average of two independent experiments. (*D*) Localization of a Rnt1p-GFP tagged version before and after a 20 min shift to 1 M NaCl. Shown is the GFP signal from a strain expressing Rnt1–GFP, or GFP alone (*bottom* panel) in the indicated salt conditions, overlapped with the blue signal (DNA; Hoechst) or the red signal from the Cy3-labeled oligodT probe. (*E*) Northern blot analysis of *BDF2* in a strain in which Rnt1p is exported to the nucleus using the anchor away technique. Shown is a northern blot of *BDF2* showing the full length (FL) and cleavage fragment (CF) in a strain expressing Rnt1p tagged with a FKBP12-rapamycin binding domain (FRB) (Haruki et al. 2008). Strains were first treated with Rapamycin or control medium for 1 h, and then shifted to 0.6 M NaCl containing medium for 1 h or maintained in a similar medium for the control samples. Numbers indicate the levels of the full-length *BDF2* relative to the *scR1* control (average of three independent replicates).

*BDF2* expression has been shown to be extensively regulated post-transcriptionally in the nucleus through two distinct pathways: spliceosome-mediated decay (SMD) (Volanakis et al. 2013) and RNase III-mediated decay (RMD) (Roy and Chanfreau 2014). During SMD, the *BDF2* mRNA undergoes the first step of splicing at a 5′ splice site sequence (5′SS, Fig. 1A), but the subsequent intermediates are released and degraded, instead of proceeding through the second splicing step (Volanakis et al. 2013). RMD is initiated by cleavage of the *BDF2* mRNA by Rnt1p, the sole representative of the RNase III family of double-stranded RNA endonucleases in *S.cerevisiae* (Elela et al. 1996; Roy and Chanfreau 2012). After cleavage in a stem–loop structure of *BDF2* by Rnt1p (the Rnt1 cleavage site or RCS, Fig. 1A), the cleavage fragments are subsequently degraded by nuclear exoribonucleases (Roy and Chanfreau 2014). The major upstream cleavage product (CF_m_, Fig. 1A) can be detected upon partial inactivation of the nuclear exosome through a deletion of its nuclear component Rrp6p (Roy and Chanfreau 2014). RMD and SMD are not completely independent, as the 5′-exon released after SMD can undergo RMD (Roy and Chanfreau 2014), resulting in a shorter cleavage fragment shown as CF_s_ on Figure 1A. Interestingly, the two major mechanisms of *BDF2* nuclear decay are activated by different environmental stresses. During osmotic stress, RMD predominates over SMD while the opposite is true during DNA replication stress (Roy and Chanfreau 2014). The increase in the activity, or hyperactivation of RMD during salt stress results in a drastic decrease of the available pool of *BDF2* transcripts and is also responsible for the extreme salt sensitivity of *bdf1*Δ mutants (Roy and Chanfreau 2014). However, the mechanism by which RMD is hyperactivated during stress remains unclear. In this study, we provide evidence that the increased cleavage of the *BDF2* transcripts during specific stress conditions is primarily due to increased retention of the *BDF2* mRNAs within the nucleus. These results show that RMD can act as an additional layer in regulating gene expression, where transcripts are retained within the nucleus and subsequently degraded by RMD.

## RESULTS

### Rnt1p protein levels remain stable in salt stress

Previous studies have shown that the *BDF2* mRNA can be regulated by both spliceosome mediated decay (SMD) (Volanakis et al. 2013) as well as RNase III-mediated decay (RMD) (Roy and Chanfreau 2014). In the presence of high concentrations of NaCl (0.6–1 M), RMD is hyperactivated and promotes degradation of the *BDF2* transcript (Roy and Chanfreau 2014). After treatment of cells with 0.6 to 1 M NaCl, the 5′-cleavage fragment generated by RMD becomes readily detectable by northern blot analysis, while the full-length *BDF2* mRNA progressively disappears (Roy and Chanfreau 2014). To further investigate the mechanism responsible for RMD hyperactivation on the *BDF2* transcript after exposure to high concentrations of NaCl, we first analyzed Rnt1p protein levels by western blot, as increased Rnt1p expression might be responsible for increased cleavage of *BDF2*. Western blot analysis of an HTP (His6-TEV-Protein A; Granneman et al. 2009)-tagged version of Rnt1p showed no significant changes in Rnt1p protein levels (paired *t*-tests *P*-values >0.1; quantifications, averages, standard deviations and statistical tests applied for all the quantitative data included in the article are shown in Supplemental Table S1) or electrophoretic mobility between samples grown in standard medium versus those treated with high salt (Fig. 1B). We verified that this HTP-tagged version of Rnt1p was functional for promoting *BDF2* RMD (Fig. 1C). This suggests that the hyperactivation of RMD of the *BDF2* transcript is not a consequence of an overall increase in Rnt1p protein levels.

### Rnt1p remains localized in the nucleus during salt stress and its nuclear localization is necessary for *BDF2* RMD

Rnt1p is primarily localized in the nucleoplasm and nucleolus in normal growth conditions (Catala et al. 2004; Henras et al. 2004). However, it is unknown if the subcellular localization of Rnt1p changes during stress or under different environmental conditions. We hypothesized that if the bulk of the *BDF2* mRNA is cytoplasmic, an increase in Rnt1p cytoplasmic localization during osmotic stress may result in an increase in *BDF2* RMD. In order to visualize the localization of Rnt1p within the cell, we utilized a strain expressing a GFP-tagged version of Rnt1p, which was previously shown to be functional (Henras et al. 2004) and which can promote *BDF2* mRNA RMD during stress (see below). We did not observe a difference in the subcellular localization of Rnt1p in strains grown in normal conditions or treated with 1 M NaCl (Fig. 1D), as the major site of GFP accumulation was still detected in the nucleus, as previously shown (Henras et al. 2004). This localization was specific as it was not detected using a strain expressing a GFP control (Fig. 1C). The primary nuclear localization of Rnt1p during salt-induced stress suggests that Rnt1p-mediated cleavage of *BDF2* transcripts during stress is likely to occur within the nucleus. Previous studies have shown that environmental stresses can cause global poly(A)^+^ mRNA retention within the nucleus (Piper 1995; Saavedra et al. 1996; Izawa et al. 2008). Thus, it is conceivable that salt stress may cause similar global nuclear retention of poly(A)^+^ mRNAs. In this scenario, nuclear retained transcripts would be more likely to undergo RMD. Using Cy3-labeled oligo d(T)_50_ to visualize polyadenylated mRNAs by fluorescence in situ hybridization (FISH), a distinct pattern of nuclear poly(A)^+^ mRNA aggregation was detected after exposure to 1 M NaCl salt stress, contrasting to the diffuse cytoplasmic pattern detected before stress (Fig. 1D). We tried to specifically detect the *BDF2* mRNAs by FISH in these conditions, but we were unable to obtain consistent data using oligonucleotide probes complementary to the *BDF2* sequence (not shown). Taken together, our data show that high salt stress does not induce an overall change in the nuclear localization of Rnt1p but results in an overall change in the localization of poly(A)^+^ mRNAs that may contribute to promoting RMD in the nucleus.

To further show that the nuclear localization of Rnt1p is necessary for *BDF2* RMD, we depleted Rnt1p from the nucleus using the anchor away technique (Haruki et al. 2008) prior to salt stress exposure. We used a strain in which Rnt1p is tagged with a *FKBP12*-rapamycin binding domain (abbreviated as FRB), which can be used to promote the rapid export of nuclear proteins to the cytoplasm upon addition of rapamycin (Haruki et al. 2008). Anchoring away Rnt1p to the cytoplasm prior to 1 h of NaCl-induced stress was sufficient to prevent *BDF2* RMD cleavage (Fig. 1E; lane 4 vs. lane 3 paired *t*-test *P*-value = 0.017), further supporting the hypothesis that RMD predominantly occurs within the nucleus. Altogether, these data indicate that Rnt1p localization remains unaltered in salt stress and that RMD is dependent on the nuclear localization of Rnt1p.

### *BDF2* RMD hyperactivation can be detected in a variety of stress conditions that are known to result in mRNA nuclear retention

**FIGURE 2.**
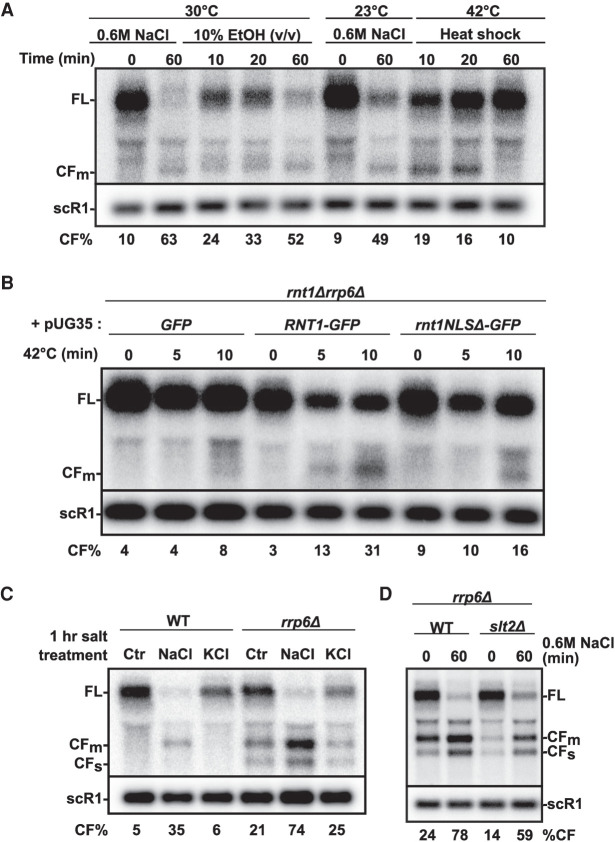
Analysis of *BDF2* RMD in various stress conditions. (*A*) Northern blot analysis of *BDF2* following exposure to ethanol (10%), heat shock (42°C), or salt stress conditions (0.6 M NaCl). scR1 was used as a loading control. The migration of the full-length (FL) and of the main cleavage fragment (CFm) is indicated. Numbers indicate the % of the *BDF2* cleavage fragments relative to all *BDF2* transcripts. The numbers shown are the average of three independent biological replicates, except for lane *5* (60 min ethanol treatment) for which only two independent replicates were obtained. (*B*) Northern blot analysis of *BDF2* in an *rrp6*Δ*rnt1*Δ strain expressing GFP alone, Rnt1–GFP or Rnt1–GFP partially delocalized to the cytoplasm (NLSΔ) from pUG35 plasmids (Henras et al. 2004) in normal and heat-shock conditions. Numbers indicate the % of the *BDF2* cleavage fragment relative to all *BDF2* transcripts calculated from the northern blot shown on the picture which is the only biological replicate for this experiment. (*C*) Northern blot analysis of *BDF2* in control medium or following a 1 h exposure to 0.6 M NaCl or 0.6 M KCl. Numbers indicate the % of the *BDF2* cleavage fragment relative to all *BDF2* transcripts (average of three independent replicates). (*D*) Northern blot analysis of *BDF2* during NaCl-induced stress in the *rrp6*Δ and *rrp6*Δ*slt2*Δ mutants. Legends as in Figure 2A. Numbers indicate the % of the *BDF2* cleavage fragment relative to all *BDF2* transcripts based on the average of three independent biological replicates.

The previous data showed that nuclear localization of Rnt1p is necessary for the cleavage of *BDF2* transcripts, suggesting that an increase of *BDF2* mRNA nuclear retention during salt stress may be a primary mechanism for RMD hyperactivation. To further test this hypothesis, we subjected *S. cerevisiae* to various stress conditions and analyzed *BDF2* transcripts by northern blotting. RMD hyperactivation during these stress conditions was visualized either by estimating the amount of full-length mRNA remaining or by calculating the overall % of cleavage fragment (CF) over the overall *BDF2* transcripts population [%CF = CF/(CF + FL) × 100]. Ethanol or heat shock stress are known to cause selective retention of bulk mRNAs within the nucleus and the rapid export of stress responsive transcripts (Piper et al. 1994; Saavedra et al. 1997; Izawa et al. 2008). Strikingly, we detected RMD hyperactivation of *BDF2* in cells treated with either ethanol or heat shock (Fig. 2A) but the kinetics and final effect on full-length *BDF2* levels varied. Ethanol treatment resulted in a global decrease of the *BDF2* mRNA and an accumulation of the main cleavage fragment CFm after 1 h. However, during heat shock the cleavage fragment was detected at its peak within the first 20 min of treatment and the level of the *BDF2* FL mRNA was not strongly affected. No cleavage fragment was detected after an hour of heat shock, as the cells may have recovered from the stress. This is consistent with RNA-seq analysis done after 45 min of heat shock (Wang et al. 2020), which showed that *BDF2* full-length levels were similarly to control samples after prolonged heat-shock. Interestingly, a complete loss of the full length *BDF2* transcript was not detected during salt treatment at 23°C, as compared to salt treatment at 30°C (compare FL between lanes 7 and 2, Fig. 2A). This suggests that the steady state growth temperature may influence the cleavage activity of Rnt1p as well. Overall, these data using heat shock and ethanol stress treatments suggest that the RMD hyperactivation seen on *BDF2* mRNAs may be due to its nuclear retention, which has been shown to occur globally on mRNAs in these stress conditions (Piper et al. 1994; Saavedra et al. 1997; Izawa et al. 2008).

To test the hypothesis that *BDF2* RMD during heat-shock requires Rnt1p nuclear localization, we analyzed *BDF2* during heat shock in a *rnt1*Δ *rrp6*Δ double deletion mutant strain expressing either GFP alone, the *RNT1*–GFP fusion used for the localization studies shown above, or a *RNT1*–GFP carrying a deletion of the nuclear localization signal of Rnt1p (NLSΔ), which results in a large fraction of Rnt1p delocalized to the cytoplasm (Henras et al. 2004). Cells expressing the Rnt1–GFP fusion exhibited a decrease of the FL form of *BDF2* after heat-shock, with an increased accumulation of the *BDF2* cleavage fragment compared to cells expressing GFP alone (Fig. 2B). This result shows that the Rnt1–GFP fusion is functional for RMD during heat shock. In contrast, cells expressing the Rnt1p–NLSΔ mutant showed a delay in the accumulation of the *BDF2* cleavage fragment, with none detectable after 5 min of heat shock treatment, and a reduced accumulation after 10 min compared to the wild-type Rnt1–GFP (Fig. 2B). RMD was not detected in the strain expressing GFP alone, showing that the band detected upon heat-shock is the result of Rnt1p cleavage. Even though this experiment was only performed once, this result shows that a fully functional Rnt1p localization signal is required for optimal RMD of *BDF2* during heat shock. These experiments also show that, as opposed to salt stress, heat shock results in a transient activation of RMD.

We further explored the type of ionic stress that can mediate RMD of *BDF2*. We found that the addition of high KCl concentrations (0.6 M) did not activate RMD, indicating that RMD hyperactivation is specific to the type of ionic stress (Fig. 2C). A similar result was obtained when nuclear exosome activity was impaired by the deletion of Rrp6p to increase detection of the CF (Fig. 2C). Altogether, these results demonstrate that *BDF2* RMD can be hyperactivated in a variety of stress conditions that are known to specifically induce mRNA nuclear retention.

### *BDF2* RMD hyperactivation is reduced in the *slt2*Δ mutant but can be uncoupled from extracellular stress by anchoring away mRNA biogenesis or export factors

Specific stress conditions, including salt stress and heat stress can promote the nuclear retention of non-heat shock mRNAs as a mechanism to rapidly change gene expression (Carmody et al. 2010). Slt2p (also referred to as Mpk1p) is a key protein kinase involved in the stress response (Kim and Levin 2011), which is required for the retention of non-heat shock mRNAs during heat shock conditions (Carmody et al. 2010). We used a strain carrying a deletion of the *SLT2* gene to determine if stress-induced hyperactivation of RMD is caused by *BDF2* nuclear retention through the Slt2p pathway. We performed this experiment in an *rrp6*Δ background to maximize the detection of the *BDF2* cleavage fragment. The absence of Slt2p significantly reduced *BDF2* RMD activation during NaCl-induced stress (lane 4 vs. lane 2; paired *t*-test *P*-value = 0.008; Supplemental Table S1), as shown by the increased accumulation of the full-length *BDF2* mRNA and the relative decrease of the cleavage product during stress compared to the wild-type control (Fig. 2D). However, the absence of Slt2p did not fully inhibit RMD activity on *BDF2* during salt stress. This result suggests that Slt2p might contribute to the increased retention of *BDF2* during stress, but that the *BDF2* mRNA could also be retained through a different pathway not defined by Slt2p. For example, specific stress signals may impact the mRNP formation, processing and export of several transcripts differently (Izawa et al. 2008).

**FIGURE 3.**
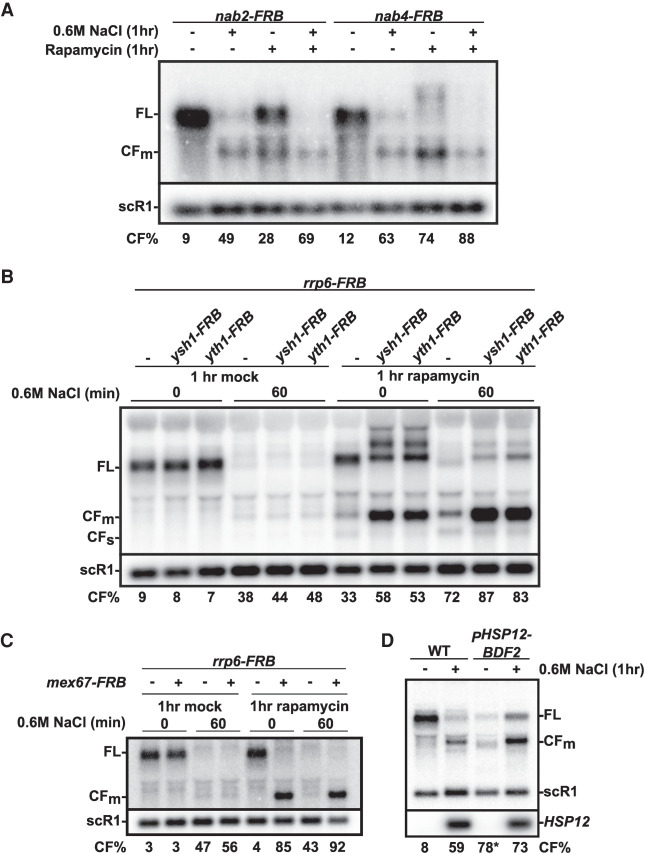
Anchoring away mRNA biogenesis or mRNA export factors triggers RMD hyperactivation of *BDF2* in the absence of stress. (*A*) Northern blot analysis of *BDF2* in strains expressing FRB-tagged versions of Nab2p, and Nab4p in salt stress conditions and/or in conditions triggering nuclear export of these factors upon addition of Rapamycin. Cells grown to log phase were split in half, with one kept in normal medium and the other half treated with 0.6 M salt. After an hour of salt treatment, each culture was split in half again and treated with either 1 µg/mL of rapamycin or its vehicle solvent (90% ethanol and 10% Tween-20) for an additional hour. scR1 was used as a loading control. The *BDF2* full-length (FL) and cleavage fragments (CFm) are shown, with the scR1 loading control. Numbers indicating the % of the *BDF2* cleavage fragment relative to all *BDF2* transcripts are the average of three independent biological replicates. (*B*) Northern blot analysis of *BDF2* in strains expressing FRB-tagged versions of Rrp6p and of the cleavage and polyadenylation factors Yth1p or Ysh1p, in salt stress conditions and/or upon addition of Rapamycin. Legends are as in Figure 3A. Numbers indicating the % of the *BDF2* cleavage fragment relative to all *BDF2* transcripts are the average of three independent biological replicates, except for lanes *1, 4, 7,* and *10* (two independent replicates), and *2* and *3* (one replicate). (*C*) Northern blot analysis of *BDF2* in a strain expressing FRB-tagged versions of Rrp6p and of the mRNA export factor Mex67p in salt stress and/or upon addition of Rapamycin. Legends are as in Figure 3A. Numbers indicating the % of the *BDF2* cleavage fragment relative to all *BDF2* transcripts are the average of three independent biological replicates. (*D*) Northern blot analysis of the *BDF2* and *HSP12* mRNAs in a wild-type strain and in a strain expressing *BDF2* from the *HSP12* promoter in normal and in salt stress conditions. Legends as in Figure 3A. Numbers indicating the % of the *BDF2* cleavage fragment relative to all *BDF2* transcripts are the average of ten (lanes *1* and *2*) or three (lanes *3* and *4*) independent biological replicates.

To further test the hypothesis that the hyperactivation of RMD on *BDF2* is primarily due to its nuclear retention during stress, we tested the effect of globally inhibiting mRNA export to the cytoplasm independently from any extracellular stress. We used the anchor-away technique (Haruki et al. 2008) to rapidly deplete from the nucleus several proteins that are involved directly or indirectly in the export of mRNAs to the cytoplasm. We first focused on the poly(A) binding protein Nab2p and on the 3′-end processing factor Nab4p (Hrp1p), as defects in poly(A) tail binding or formation have been shown to inhibit mRNA export (Hammell et al. 2002). As a control, salt treatment of strains in which Nab2p and Nab4p were FRB-tagged (but without adding rapamycin which promotes the anchor away process) resulted in a reduction of the full length *BDF2* transcripts and in the appearance of the cleavage product CFm (Fig. 3A). Strikingly, cleavage of *BDF2* transcripts was detected in the absence of any salt stress after anchoring away Nab2p or Nab4p (Fig. 3A). The size of the fragment detected upon anchoring away Nab2p or Nab4p in the absence of stress was identical to the size of the fragment detected during salt stress (Fig. 3A), which showed that RMD was activated upon anchoring away Nab2p or Nab4p. The extent of *BDF2* cleavage differed, with Nab4p resulting in a stronger accumulation of the CFm and in a very strong reduction in the levels of the FL form of *BDF2*; however, this reduction was not solely due to RMD, as long extended forms could also be detected due to transcription termination defects arising from defective 3′-end formation when Nab4p is inactivated (Minvielle-Sebastia et al. 1998). Nevertheless, these results suggested that the inhibition of mRNA export triggered by defects in poly(A) formation or binding is sufficient to recapitulate the salt stress-induced RMD hyperactivation of *BDF2*. However, anchoring away Nab2p or Nab4p did not result in an activation of RMD to the same extent as that detected during salt stress, and a further loss of the full-length transcript was observed when cells were exposed to salt stress prior to Rapamycin treatment (Fig. 3A). This additive effect may either indicate a synergy between the retention of unprocessed RNAs and the retention of mature RNAs during stress for RMD activation, or that the anchoring away of either of these two factors may not completely abolish mRNA export.

To further explore the hypothesis that inhibiting 3′-end processing can result in *BDF2* RMD hyperactivation, we constructed strains in which the core cleavage and polyadenylation (CPA) factors Yth1p and Ysh1p can be anchored away from the nucleus, concomitantly with Rrp6p (Fig. 3B). This double anchor away strategy was used to allow a better detection of the *BDF2* cleavage product as anchoring away Rrp6p strongly stabilizes nuclear exosome targets (Roy et al. 2016), possibly because other subunits of the exosome might be anchored away concomitantly. Strikingly, anchoring away both Rrp6p and Ysh1p or Yth1p resulted in a strong accumulation of the *BDF2* cleavage product (Fig. 3B) in the absence of any extracellular stress. The fragment detected upon anchoring away Ysh1p or Yth1p and Rrp6p in the absence of stress comigrated with the fragment detected during salt stress treatment alone (Fig. 3A), which showed that this fragment was generated by Rnt1p cleavage. The accumulation of the *BDF2* cleavage fragment and the decrease of the full-length mRNA were exacerbated when strains were exposed to salt stress prior to anchoring away these CPA factors. Similar to what was observed with Nab4p, anchoring away Ysh1p and Yth1p also resulted in the appearance of extended species of *BDF2*, likely because of the transcription termination defects resulting from CPA inactivation. These results show that inactivation of different CPA factors can promote the hyperactivation of *BDF2* RMD.

To formally demonstrate that inhibition of mRNA nuclear export can trigger RMD hyperactivation, we performed nuclear depletion of the essential mRNA export factor, Mex67p concomitantly with Rrp6p. Mex67p is a core member of the mRNA export complex (Hurt et al. 2000) and its nuclear depletion through the anchor-away technique completely abolishes poly(A)^+^ mRNA export (Haruki et al. 2008). Strikingly, co-nuclear depletion of Mex67p and Rrp6p fully reproduced *BDF2* RMD hyperactivation (Fig. 3C) in the absence of extracellular stress. Nuclear depletion of Mex67p resulted in a complete loss of the full length *BDF2* transcripts, a phenotype stronger than the nuclear depletion of Nab2p or of the 3′-end processing factors Nab4p, Yth1p, or Ysh1p. The complete cleavage of the *BDF2* transcripts was further confirmed by the observation that no additional cleavage product accumulated when the anchored-away strains were treated with salt stress (Fig. 3C). Overall, these results indicate that the subcellular localization of *BDF2* mRNAs plays a pivotal role in determining their degradation fate through RMD, and that inhibition of mRNA nuclear export by triggering export of key mRNA biogenesis or export factors can fully recapitulate RMD hyperactivation of *BDF2* in the absence of extracellular stress.

### Promoter identity is not required to promote *BDF2* RMD during stress

Elements within gene promoters can affect the stability of mRNAs expressed from these promoters (Trcek et al. 2011; Catala and Abou Elela 2019). Promoter-dependent regulation of mRNA stability is controlled through the cotranscriptional recruitment of several proteins that initiate mRNA decay. Rnt1p itself can be recruited to the promoter before recognizing the Rnt1p cleavage site (RCS) within the ORF (Catala and Abou Elela 2019). To determine if *BDF2* RMD relies on the recruitment of Rnt1p to the *BDF2* promoter, we swapped the endogenous *BDF2* promoter with that of *HSP12.* The *HSP12* mRNA does not undergo RMD, and we hypothesized that this promoter would be unable to recruit Rnt1p despite containing many stress response elements (STRE) (Varela et al. 1995). Furthermore, the *HSP12* promoter is strongly activated under salt stress, allowing us to investigate the RMD-mediated regulation of the *BDF2* transcript generated from this promoter (*_p_HSP12-BDF2)*. The *BDF2* mRNA generated from *_p_HSP12-* was still targeted by Rnt1p for rapid cleavage under salt stress (Fig. 3D). In fact, the strong induction of the *HSP12* promoter under salt stress generated more transcripts to be targeted by RMD, resulting in an increase of accumulation of the cleavage fragment compared to wild type post-salt stress. RMD was however not sufficient to completely eliminate the highly expressed *_p_HSP12-BDF2* transcript, possibly due to the known ability of STRE to promote rapid export in heat shock through recruitment of the mRNA export factor Mex67p by Hsf1p (Zander et al. 2016). We conclude that the RMD hyperactivation on the *BDF2* transcript does not require recruitment of Rnt1p to the endogenous *BDF2* promoter.

### The identity of the stem–loop cleaved by Rnt1p influences RMD efficiency during stress

**FIGURE 4.**
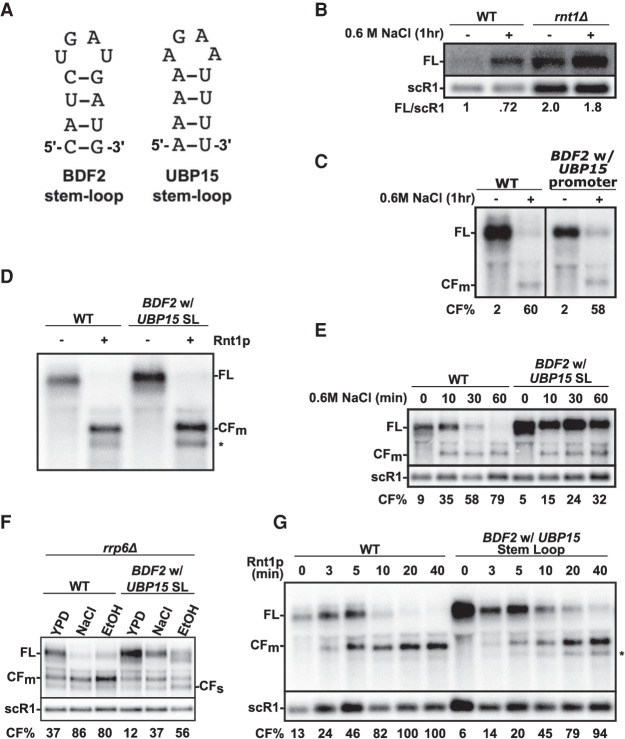
The identity of the Rnt1p cleavage site (RCS) influences RMD hyperactivation in stress conditions. (*A*) Predicted secondary structures of the terminal stem–loops present at the top of the RCS of *BDF2* and *UBP15* using Mfold (Zuker 2003). (*B*) Analysis of *UBP15* mRNA expression in WT and *rnt1*Δ cells in normal growth conditions and upon salt stress treatment. Shown is a northern blot analysis of *UBP15* in the corresponding strains. scR1 was used as a loading control. This experiment was performed once and the ratios indicate the level of full-length *UBP15* relative to scR1 based on the northern blot shown. (*C*) Northern blot analysis of *BDF2* in normal or NaCl-stress conditions for the endogenous *BDF2* gene or the gene expressing *BDF2* from the *UBP15* promoter. The FL and main cleavage fragments are indicated. This experiment was performed once and the %CF was calculated from the northern blot shown on this figure. (D) In vitro cleavage assay of *BDF2* transcripts containing the RCS of *UBP15.* Shown is a northern blot of total RNAs from the corresponding strains after incubation in buffer or with recombinant Rnt1p for 1 h. The location of the main cleavage fragment (CFm) is indicated. The asterisk indicates a secondary, minor cleavage event detected for the *BDF2*–*UBP15* SL substrate. (*E*) Northern blot of wild-type *BDF2* and *BDF2* transcripts harboring the RCS from *UBP15* after exposure to 0.6 M NaCl stress. Legends as in Figure 2. Numbers indicating the % of the *BDF2* cleavage fragment relative to all *BDF2* transcripts are the average of three independent biological replicates. (*F*) Northern blot of *BDF2* in an *rrp6*Δ mutant expressing the wild-type *BDF2* or a *BDF2* mutant with the *UBP15* stem–loop in normal medium, or after exposure to 0.6 M NaCl or 10% ethanol for 1 h. Numbers indicating the % of the *BDF2* cleavage fragment relative to all *BDF2* transcripts are the average of three independent biological replicates. (*G*) Time course of in vitro cleavage of the wild-type *BDF2* and *BDF2* RNA with the *UBP15* terminal stem–loop by recombinant Rnt1p. Total RNAs extracted from the wild-type strain or from a strain expressing *BDF2* with the *UBP15* terminal stem–loop were incubated with recombinant Rnt1p for the indicated times. The reactions were stopped, the RNAs repurified and analyzed by northern blot. The numbers indicate the % of *BDF2* cleavage [(CF/CF + FL) × 100] relative to time zero for each substrate for the time course experiment shown on this figure.

Previous studies have demonstrated that specific structural features of the stem–loop recognized by Rnt1p may affect cleavage efficiency (Comeau et al. 2016). To further investigate how the stem–loop present in *BDF2* may affect RMD hyperactivation*,* we replaced the endogenous *BDF2* RCS with that of *UBP15* (Fig. 4A). The *UBP15* mRNA does not undergo RMD hyperactivation in salt stress (Fig. 4B) and replacing the *BDF2* promoter by the *UBP15* promoter does not abrogate RMD (Fig. 4C), which showed that the lack of cleavage of *UBP15* in vivo during stress is not due to its endogenous promoter, which could have promoted its export from the nucleus as shown for other stress induced genes (Zander et al. 2016). However, the *BDF2* mRNA containing the stem–loop (SL) present in *UBP15* was fully cleaved when incubated for 1 h with recombinant Rnt1p in vitro (Fig. 4D). Incubation of total RNAs containing the *BDF2* hybrid transcripts containing the *UBP15* SL with recombinant Rnt1p resulted in detection of a cleavage product with a migration identical to that detected upon in vitro cleavage of the wild-type *BDF2* (Fig. 4D). This indicates that the hybrid *BDF2* transcripts can be indeed targeted and cleaved by Rnt1p in the same stem–loop structure. We note that the *UBP15* terminal loop also induced the cleavage at an additional, minor site (asterisk on Fig. 4D). Based on these in vitro cleavage data, it is unclear whether the absence of RMD activity on *UBP15* in vivo is due to the identity of its RCS or to other factors that might prohibit cleavage in vivo. To further investigate the impact of the RCS identity on RMD, we analyzed the levels of wild-type *BDF2* and of the mutant containing the *UBP15* SL during stress. The *BDF2* hybrid transcript containing the *UBP15* SL was less efficiently down-regulated by RMD during a kinetic of salt exposure (Fig. 4E), with significant decreases of RMD efficiency compared to the WT based on the %CF (all time points paired *t*-tests *P*-values <0.05; Supplemental Table S1). To better characterize the impact of the *UBP15* stem–loop on *BDF2*, we introduced this mutation in the context of the *rrp6*Δ mutant, and we analyzed *BDF2* levels in control medium (YPD) or after 1 h of 0.6 M NaCl or 10% thanol treatment (Fig. 4F). In rich medium, the presence of the *UBP15* SL resulted in a substantial increase of the FL form and a decrease of both CFm and CFs (Fig. 4F), reflecting a significant decrease in Rnt1p cleavage activity in normal growth conditions (lane 1 vs. 4; *t*-test *P*-value = 0.005; Supplemental Table S1). The *UBP15* stem–loop also decreased the hyperactivation of *BDF2* RMD during stress, based on the %CF ratios indicated and as shown by a substantial amount of the full length *BDF2* hybrid transcript remaining after an hour of salt or ethanol treatment compared to the wild-type *BDF2* (paired *t*-test *P*-values: NaCl treatment = 0.001; ethanol treatment = 0.05). To further characterize the impact of the *UBP15* stem–loop on Rnt1p cleavage activity, a kinetic analysis of in vitro cleavage was performed using purified Rnt1p and total RNAs as a source of substrate RNA. As shown in Figure 4G the rate of in vitro cleavage of the hybrid transcript was slower than that observed for the wild-type *BDF2* transcript (Fig. 4G). Although this time course experiment was performed only once, the decreased cleavage efficiency is consistent with the decreased cleavage activity detected in vivo in Figure 4E,F. Inefficient cleavage of this substrate compared to the WT could be due to the fact that the UBP15 stem–loop is rich in A–U base pairs near the terminal tetraloop, which is unusual for Rnt1p substrates (Chanfreau 2003). Taken together, our data demonstrate that specific features of the RCS can affect RMD activity during normal and stress conditions and that optimal degradation of *BDF2* during stress requires both its specific stem–loop structure and inhibition of RNA export, but is not dependent on expression of *BDF2* from its natural promoter.

## DISCUSSION

Bdf2p is a bromodomain protein involved in multiple aspects of epigenetic control through its ability to recognize acetylated histones. We previously showed that the *BDF2* transcript is subject to both RMD and SMD pathways which limit and regulate its expression (Roy and Chanfreau 2014). This regulation is of particular importance during stress conditions, where a rewiring of transcription and translation is necessary to promote cellular fitness during stress. In high salt stress, an increase in activity of RMD on *BDF2* causes its transcript to become completely undetectable. Repression of *BDF2* in stress conditions is necessary for the robust expression of the stress responsive gene *GPH1*, and potentially other stress responsive genes as well (Roy and Chanfreau 2014). Despite the significance of regulating the expression of *BDF2*, the mechanism that triggers RMD hyperactivation during salt stress was not clearly understood. In this study, we demonstrate that the increase of RMD activity on *BDF2* during stress does not arise from a direct change in the expression of Rnt1p or its localization within the cell. Rather, our study suggests that the nuclear retention of *BDF2* transcripts during stress causes RMD hyperactivation. A global block of nuclear export of mRNAs induced by stress conditions such as heat or ethanol shock, or by nuclear depletion of specific mRNA biogenesis or export factors, can reproduce salt stress-induced RMD hyperactivation on *BDF2*. We also show that inactivation of the key stress response factor Slt2p reduces the efficiency of *BDF2* RMD during stress. We interpreted this reduction in RMD in the *slt2*Δ mutant as a consequence of the role of Slt2p in mediating stress-induced mRNA nuclear retention (Carmody et al. 2010). However, we do not know if the reduction of RMD efficiency detected in this mutant for RMD is dependent on its kinase and signaling activity, as Slt2p has kinase-independent roles during stress (Kim and Levin 2011). Taken together, these results show that the primary mechanism underlying the hyperactivation of RMD for *BDF2* is the inhibition of mRNA export and the retention of transcripts in the nucleus. RMD hyperactivation is independent of the identity of the *BDF2* transcript promoter region, as switching its promoter to that of *HSP12* or *UBP15* does not prevent RMD. This suggests that promoter-dependent recruitment of Rnt1p, which is required for RMD of other transcripts (Catala and Abou Elela 2019) does not play a role in the hyperactivation of RMD of *BDF2* observed in stress conditions.

We further provide evidence that the identity of the Rnt1p cleavage stem–loop (RCS) within *BDF2* further influences its susceptibility to cleavage, as replacing the endogenous *BDF2* terminal stem–loop by that of *UBP15* decreases *BDF2* RMD in vivo, and Rnt1p cleavage efficiency in vitro. This supports previous evidence that the nucleotide base-pairing of the product termini can determine the Rnt1p substrate reactivity (Comeau et al. 2016). The effect of the *UBP15* stem–loop on Rnt1p cleavage might be linked to its stretch of A–U base pairs near the terminal tetraloop (Fig. 4A), which is not usually found in natural Rnt1p substrates (Chanfreau 2003). It is unclear whether certain stress conditions, such as heat shock or salt stress, may alter the structure of the RCS in vivo and influence its cleavage efficiency by Rnt1p. High temperatures or specific salt conditions may contribute to the stabilization or destabilization of the *BDF2* RCS and thereby modulate cleavage efficiency. This may contribute to explain some of the differences in *BDF2* RMD efficiency detected between heat shock treatment, ethanol or salt stress. Alternatively, it is possible that these different stress conditions affect mRNA export out of the nucleus differently, or that they could impact degradation pathways which facilitate the detection of the *BDF2* cleavage products.

Altogether, our results indicate that Rnt1p may help regulate expression of specific genes through the cleavage and degradation of specific substrates based on their localization and stem–loop structures. The nuclear export of many mRNAs is inhibited during stress, which provides the opportunity for these mRNAs to be targeted by Rnt1p for degradation in these conditions to differing extents, depending on substrate reactivity. Previous studies have shown that blocking the nuclear export of mRNAs by nuclear depletion of Mex67p results in the rapid degradation of newly synthesized RNAs (Tudek et al. 2018). A similar effect was observed for polyadenylated RNAs when the poly(A) binding protein Nab2p was depleted from the nucleus (Schmid et al. 2015). Strikingly, anchoring away both of these factors is sufficient to promote *BDF2* RMD in the absence of stress (Fig. 3). Therefore, it is possible that Rnt1p may play a role in the general degradation mechanism previously described by the Jensen group upon nuclear depletion of these factors, and that Rnt1p-mediated cleavage may contribute to the elimination of specific mRNAs containing compatible cleavage sites during stress-induced nuclear retention of mRNAs. However, not all mRNAs sequestered in the nucleus during stress or after nuclear depletion of CPA or export factors contain stem–loop structures compatible with Rnt1p cleavage, and it is likely that the sequence content of mRNAs has evolved to either promote RMD of mRNAs that are not required or perhaps detrimental during stress conditions.

## MATERIALS AND METHODS

### Yeast strains and plasmids

All strains used in this study were derived from BMA64, HHY168 or as described below:

- Strains previously described or from external sources:
  BMA64 (Chanfreau et al. 1998): MATa; ura3-1; trp1Δ2; leu2-3;112; his3-11,15; ade2-1; can1-100
  *rnt1*Δ: BMA64, *rnt1Δ::TRP* (Chanfreau et al. 1998)
  BY4742: MATα his3Δ1 leu2Δ0 lys2Δ0 ura3Δ0 (Open Biosystems)
  HHY168 (Haruki et al. 2008) MATalpha tor1-1 fpr1::NAT RPL13A-2×FKBP1 2::TRP1 ade 2-1 trp1-1 can1-100 leu2-3,112 his3-11,15 ura3 GAL psi+ (Haruki et al. 2008)
  yCW2: MATa his3Δ1 leu2Δ0 met15Δ0 ura3Δ0 RNT1-HTTP::KIURA3 (gift of D. Tollervey).
  yCW6: BY4742, *rrp6Δ:: hphMX6* (Wang et al. 2020)
  yCW17: yCW6, *slt2Δ::kanMX6* (Wang et al. 2020)
- Strains generated in this study:
  yCW4: MATalpha tor1-1 fpr1::hphMX6 RPL13A-2×FKBP12::TRP1 ade 2-1 trp1-1 can1-100 leu2-3,112 his3-11,15 ura3 GAL psi+
  yCW5: yCW4 with *RNT1-FRB::kanMX6*
  yCW7: yCW4 with *NAB2-FRB::kanMX6*
  yCW8: yCW4 with *NAB4-FRB::kanMX6*
  yCW9: yCW4 with YTH1-FRB::kanMX6 RRP6-FRB::his3MX6
  yCW10: yCW4 with YSH1-FRB::kanMX6 RRP6-FRB::his3MX6
  yCW11: yCW4 with MEX67-FRB::kanMX6 RRP6-FRB::his3MX6
  yCW12: BMA64 with *pHSP12-BDF2*
  yCW13: BMA64 with BDF2 with *UPB15 SL*
  yCW14: BMA64 with BDF2 with UPB15 SL rrp6Δ::hphMX6
  yCW18: BMA64 rnt1Δ::TRP, rrp6Δ::hphMX6
  yCW19: BMA64 with *BDF2* with *UBP15* promoter

Strains were constructed using a high efficiency transformation method (Gietz and Schiestl 2007). Mutations within the *BDF2* transcripts were constructed through the *delitto perfetto* approach (Storici and Resnick 2006). The Rnt1p target stem–loop within the *BDF2* transcript (ChrIV:332367-80) was replaced with the CORE integration cassette, consisting of the *URA3* and *KanMX6* genes. Successful transformants were selected through their resistance to G418, and further confirmed through PCR. Afterwards, the CORE integration cassette was replaced with various Rnt1p target stem–loops using the transformation protocol as described above. Successful transformants were initially selected on the basis for their ability to grow on 5-Fluoroorotic acid (5-FOA) due to the loss of *URA3,* inability to grow on G418 due to the loss of *kanMX6*, and then further confirmed through PCR and Sanger sequencing (Laragen, Inc.).

Anchor away strains were created in a modified HHY168 (Haruki et al. 2008) background where the *natMX6* marker was replaced with the *hphMX4* marker amplified from pAG32 (Goldstein and McCusker 1999). The genes of interest were carboxy-terminally tagged with the rapamycin binding domain (FRB) using the transformation method as described. Plasmids expressing Rnt1–GFP and Rnt1–GFP with a deletion of the nuclear localization sequence are described in Henras et al. (2004).

### Oligonucleotides used for mutagenesis (F and R stand for forward and reverse primers)

BDF2 Promoter Core oligonucleotides:

F:AGAGGCGAAAAAAGAGTGCAACGTCAACAACGCTAAAAGAGAGCTCGTTTTCGACACTGG

R:AGCCGCCGAGGTTTATTTCGCTCAATCTGTTTGTTTCAGTTCCTTACCATTAAGTTGATC

HSP12::BDF2 promoter swap:

F:AATGGAGTGAAGCAGGCAGGGTGACCCTCTAGCTAAAAAA GATCCCACTAACGGCCCAGC; R:CAGAATGTGCGTGTCTTGTATCCATGTTAGTACGAGACAT TGTTGTATTTAGTTTTTTTT

UBP15::BDF2 promoter swap:

F:AGAGGCGAAAAAAGAGTGCAACGTCAACAACGCTAAAAGA CAAGAGAGCAGTAGTAAGAG

R:CAGAATGTGCGTGTCTTGTATCCATGTTAGTACGAGACAT TGTTTGTTTGAAGAGACTAA

BDF2 Rnt1 cleavage site Core insertion:

F:GGATTCAGATCTTGAAGAGGATAACTATTCTTCTTCATATGAGCTCGTTTTCGACACTGG

R:TAGTTATATCGTTTTCATTTATGTCTTCATCATCATATTCTCCTTACCATTAAGTTGATC

UBP15 stem–loop swap:

R:ATATTTTGAATGAAATTGAAACGGATTCAGATCTTGAAGAGGATGCAAAATTTGGTCTCGGACAAAAAGAATTTTCAAAG

F:TTTGTTCCAAATATTGAATAGCCGGATTAGTTATATCGTTTTCATTACTTAATTTGATCTTTGAAAATTCTTTTTGTCCG

### Yeast media and growth conditions

All strains were grown in YPD (1% yeast extract, 2% peptone, and 2% dextrose) or minimal media (0.67% w/v yeast nitrogen base, 2% w/v dextrose, and 0.2% w/v amino acid mixture) at 30°C unless noted otherwise. For protein and RNA extractions, 50 mL of culture were harvested at OD_600_ 0.4–0.6 by centrifugation at 4000 rpm (Sigma Rotor 11030) for 1.5 min and transferred to 2 mL screw capped Eppendorf tubes. The cells were pelleted and flash frozen in liquid nitrogen. For experiments involving treatment involving anchor away and salt stress conditions, specific conditions are described in the results or legends of each figure. For heat shift experiments, cells were grown to exponential phase at 23°C before equal volumes of 61°C preheated YPD were added to bring the temperature to 42°C. The cultures were immediately harvested at the indicated times. For ethanol treatment, cells were grown to exponential phase before equal volumes of YPD or YPD containing 20% ethanol (v/v) were added, and then harvested at the indicated times.

### Yeast RNA extraction and northern blot analysis

For most experiments, northern blot analysis was performed in triplicate (unless indicated otherwise) on purified total RNAs as described previously (Roy and Chanfreau 2014). Briefly, RNAs were extracted using phenol-chloroform, precipitated with ethanol and sodium acetate, and resuspended in water. A total of 5 µg of glyoxylated RNAs were run on a 1.8% agarose gel and transferred to nylon membranes for probing.

### Riboprobe and oligonucleotide probe synthesis for northern blotting analysis

The radiolabeled riboprobes were transcribed in vitro using T3 RNA polymerase (Promega) as described previously (Roy and Chanfreau 2014). The *BDF2* riboprobe hybridizes from the beginning of the open-reading frame until the Rnt1p cleavage site.

Templates used were synthesized by PCR using the primers: BDF2 F: GCACATTCTGCTTTACTGGCAGC and BDF2 T3R: GGCTAAATTAACCCTCACTAAAGG TTCAAGATCTGAATCCGTTT.

The SCR1 Oligonucleotide used for the loading control: ATCCCGGCCGCCTCCATCAC was radiolabeled using γ-^32^P-ATP (PerkinElmer) and T4 polynucleotide kinase (New England Biolabs) according to the manufacturer's protocol.

### In vitro Rnt1p cleavage assay

Recombinant Rnt1p was purified as described previously (Henras et al. 2005). In vitro cleavage reactions were performed in 50 µL reactions consisting of 50 µg of total yeast RNA, 10 pmol of purified recombinant Rnt1p in Rnt1p cleavage buffer (30 mM Tris pH 7.5, 150 mM KCl, 5 mM spermidine, 200 mM MgCl_2_, 0.1 mM DTT, 0.1 mM EDTA). The reactions were incubated at 30°C and halted by the addition of 150 µL of RNA buffer at the times indicated. The reactions were then purified through using phenol-chloroform. Briefly, 200 µL of phenol: chloroform: isoamyl alcohol were added to the samples. The samples were vortexed for 1 min and spun down for 2 min at 15,000 rpm. The top aqueous layer was added to a fresh Eppendorf tube containing 1 mL ethanol, 40 µL 3 M sodium acetate pH 5.2, and 1 µL of GlycoBlue (Ambion). Precipitation of the RNA was facilitated through incubating the samples at −80°C for 30 min and then pelleted by centrifugation at 15,000 rpm for 10 min. The pellets were washed with 200 µL of 70% ethanol and resuspended in 15 µL of nuclease-free water.

### Protein extraction and western blot analysis

Western blot analysis was performed using protein extracts from an Rnt1p-HTP strain kindly provided by D. Tollervey (U. Edinburgh). The HTP carboxy-terminal tag (Granneman et al. 2009) consists of a 6-His sequence, followed by a TEV protease cleavage site and a Protein A sequence (PMID: 19482942). Total protein was prepared from the Rnt1p-HTP strain and an untagged control strain grown to mid-log phase in 50 mL YPD liquid medium and treated with 0.6 M NaCl for 0, 15, and 60 min. The culture was harvested by centrifugation for 5 min at 3500 rpm, washed with ddH_2_O, and resuspended in lysis buffer (200 mM Tris-HCl pH 8.0; 320 mM Ammonium sulfate; 5 mM MgCl_2_; 10 mM EGTA pH 8.0; 20 mM EDTA pH 8.0; 1 mMDTT; 20% glycerol; 1 mM PMSF; 2 mM benzamidine HCl and protease inhibitor cocktail). The sample was vortexed with glass beads (roughly equivalent volume to size of cell pellet) for 8 min at 4°C. Supernatant was collected by centrifugation at max speed (13.2k rpm) for 5 min at 4°C. Total protein concentration was measured using the Bradford method with the Bio-Rad Protein Assay (#500-0006) according to the manufacturer's protocol. A total of 10 µg total protein from crude extracts was analyzed by 8% SDS-PAGE and transferred to PVDF membranes for western blot analysis. Rnt1p-HTP was detected with anti-protein A antibody conjugated to horseradish peroxidase (HRP) (Invitrogen PA1-26853) diluted 1:8000 in blocking solution (1× PBS-T, 5% milk) with the Pierce ECL Western Blotting Substrate (Thermo Fisher #32209) according to the manufacturer's protocol.

### Microscopy

Strains expressing GFP-tagged Rnt1p (Henras et al. 2004) were grown to mid-log phase in standard growth conditions and treated with 0.6 M or 0.1 M NaCl for 20 min before being prepared for imaging via the Stellaris RNA FISH protocol for *S. cerevisiae* with the following modifications: All centrifugation was performed at 850*g* for 6 min instead of 400*g* for 5 min, and Hoescht stain was used instead of DAPI for nuclear staining. The FISH probe used for visualization of mRNA localization was an oligo-d(T)25 labeled with Cy3 (MilliporeSigma). Microscopy was performed on a Leica DMI4000B Confocal microscope. All images were taken with identical settings and with a *z*-stack of six images with 1 µm steps, from which one *z*-slice was chosen from each stack for further processing. Image processing was then performed and equally applied to all images with the ImageJ software.

### Reproducibility and statistics

In general, most experiments were performed in triplicate with the exception of the experiments presented in Figures 2B, 4B–D,G which included only one replicate, and some specific lanes of some figures (see figure legends). The quantification of the western blot and northern blot signals for experiments shown in all figures and for all replicates is included in Supplemental Table S1, with the calculation of the average and the standard deviations. Paired *t*-test *P*-values are included in Supplemental Table S1 for the comparisons of averages corresponding to specific lanes for each experiment.

## SUPPLEMENTAL MATERIAL

Supplemental material is available for this article.

## Supplementary Material

Supplemental Material
