# Supplementary material for: Stress-induced inhibition of mRNA export triggers RNase III-mediated decay of the BDF2 mRNA
Source: RNA. 2021 Dec;27(12):1545–56. doi: 10.1261/rna.078880.121 (PMC8594472; doi:10.1261/rna.078880.121)
Supplement: Supplemental Material [file supp_27_12_1545__DC1.html]

Stress-induced inhibition of mRNA export triggers RNase III-mediated decay of the BDF2 mRNA — Stress-induced inhibition of mRNA export triggers RNase III-mediated decay of the BDF2 mRNA — Supplemental Material 

# Stress-induced inhibition of mRNA export triggers RNase III-mediated decay of the *BDF2* mRNA

## Supplemental Material

- Supplemental\_TableS1.xlsx
